# Galectin-3 Enhances Migration of Minature Pig Bone Marrow Mesenchymal Stem Cells Through Inhibition of RhoA-GTP Activity

**DOI:** 10.1038/srep26577

**Published:** 2016-05-24

**Authors:** Qian Gao, Ying Xia, Lan Liu, Lei Huang, Yang Liu, Xue Zhang, Kui Xu, Jingliang Wei, Yanqing Hu, Yulian Mu, Kui Li

**Affiliations:** 1 State Key Laboratory of Animal Nutrition and Key Laboratory of Farm Animal Genetic Resources and Germplasm Innovation of Ministry of Agriculture, Institute of Animal Sciences, Chinese Academy of Agricultural Sciences, Beijing 100193, China

## Abstract

Bone marrow mesenchymal stem cells (BM-MSCs) are used in tissue engineering because of their migration characters. However, BM-MSCs have limitations in terms of reaching injuries and self-renewal. Therefore, enhancement of BM-MSC migration is important for therapeutic applications. Here, we assessed whether galectin-3 (Gal-3) increases the migration of minature pig BM-MSCs. Gal-3 was knocked down by short hairpin RNA (shRNA) or overexpressed using a lentiviral vector in Wuzhishan minature pig BM-MSCs. Proliferation and migration assays showed that knockdown of Gal-3 impaired BM-MSC proliferation and migration, whereas Gal-3 overexpression promoted these behaviors. RhoA-GTP activity was upregulated in Gal-3 shRNA-transfected BM-MSCs, while Rac-1- and Cdc42-GTP showed no changes. Western blotting indicated downregulation of p-AKT (ser473) and p-Erk1/2 after serum starvation for 12 h in Gal-3-knockdown BM-MSCs. p-AKT (ser473) expression was upregulated after serum starvation for 6 h, and p-Erk1/2 expression was unchanged in Gal-3-overexpressing BM-MSCs. Treatment with C3 transferase or Y27632 enhanced migration, whereas Gal-3 knockdown impaired migration in treated cells. These results demonstrate that Gal-3 may enhance BM-MSC migration, mainly through inhibiting RhoA-GTP activity, increasing p-AKT (ser473) expression, and regulating p-Erk1/2 levels. Our study suggests a novel function of Gal-3 in regulating minature pig BM-MSC migration, which may be beneficial for therapeutic applications.

Mesenchymal stem cells (MSCs) are adult stem cells characterized by their ability to self-renew and undergo multi-lineage differentiation. Bone marrow mesenchymal stem cells (BM-MSCs)^1^ migrate to injury sites and regulate repair processes^2^. BM-MSCs have been extensively applied to tissue engineering and regenerative medicine^3^. BM-MSC therapy may have benefits for various diseases such as heart infarction, Alzheimer’s disease, rheumatoid arthritis, and cardiovascular disease^4^,^5^,^6^,^7^. However, MSC numbers in the bone marrow are low, and BM-MSCs have limitations in terms of reaching injuries and self-renewal^8^,^9^. Therefore, enhancing BM-MSC migration is important. Over the past decades, MSCs have been used to understand tissue repair. Although migration towards damaged regions is necessary for MSC-mediated tissue repair, the exact mechanism requires further research.

Galectin-3 (Gal-3) is a regulator of cell migration. It is involved in various diseases including bladder transitional cell carcinoma^10^, lung cancer^11^, dysfunctional immune responses^12^, and heart failure^13^. Furthermore, a previous study has indicated that Gal-3 induces HeLa cell migration^14^. Gal-3 might be a potential biomarker to evaluate the progression of osteosarcoma and discharged heart failure patients^15^,^16^. It has been proposed to participate in multiple biological activities such as cell growth, differentiation, apoptosis, adhesion, migration, and immune activities^17^,^18^. In addition to Gal-3, glycogen synthase kinase-3β, neuritin, and erythropoietin are associated with migration of human and mouse BM-MSCs^19^,^20^,^21^. However, no study has examined the role of Gal-3 in minature pig BM-MSC migration. RhoA is highly expressed in various cell types and mainly involved in stress fiber formation and focal adhesion complex assembly. Recent reports indicate that RhoA is closely associated with cell migration, and RhoA-GTP activity affects cell migration^22^,^23^,^24^,^25^. Nevertheless, the precise relationships between Gal-3, RhoA-GTP activity, and minature pig BM-MSC migration have yet to be elucidated.

To clarify whether Gal-3 influences minature pig BM-MSC proliferation and migration, we first established stable minature pig BM-MSC lines with Gal-3 knockdown or overexpression. In these cell lines, we examined the underlying mechanism of migration enhancement by detecting changes in RhoA-GTP activity as well as p-AKT (ser473) and p-ERK1/2 levels. Our findings suggest a novel function of Gal-3 in regulating minature pig BM-MSC migration. Gal-3 might be a potential target for clinical applications of BM-MSCs. Our data provide a new perspective for MSC therapeutic strategies for tissue repair and regenerative medicine in the future.

## Materials and Methods

### Ethics statement

The present study was conducted in accordance with the recommendations in the Guide for the Care and Use of Laboratory Animals of the Chinese Academy of Agricultural Sciences (Beijing, China). The protocol was approved by the Committee on the Ethics of Animal Experiments of the Institute of Animal Science, Chinese Academy of Agricultural Sciences.

### Culture of miniature pig BM-MSCs

BM-MSCs were harvested from the femurs and tibias of 42-day-old Wuzhishan miniature pigs. The BM-MSCs were cultured in Dulbecco’s modified Eagle’s medium/F12 (Gibco) containing 10% fetal bovine serum, 1% penicillin/streptomycin, and 1% glutamine (Gibco) at 37 °C with 5% CO_2_ and 100% humidity. The medium was changed every 2 days. BM-MSCs were characterized by flow cytometric analysis as well as adipogenic and osteogenic differentiation as described previously^26^.

### Gal-3 short hairpin RNA (shRNA), lentiviral vector construction, transfection, and assessment

Based on the miniature pig Gal-3 gene sequence (GenBank number: NM_001097501.1), an RNA interference vector (Supplementary Fig. S1) and four specific shRNAs for Gal-3 were designed and synthesized by Shanghai GenePharma Co., Ltd (Galectin-3-sus-372: GGACCACTGAATGTGCCATATTTCAAGAGAATATGGCACATTCAGTGGTCCTT; Galectin-3-sus-556: GCAATTCCAAGCTGGATAATATTCAAGAGATATTATCCAGCTTGGAATTGCTT; Galectin-3-sus-444: GGCACAGTAAAGCCCAATGCATTCAAGAGATGCATTGGGCTTTACTGTGCCTT; Galectin-3-sus-738: GGGAATTTCTGGTGACATAACTTCAAGAGAGTTATGTCACCAGAAATTCCCTT). A negative control was produced according to the same design principle for shRNA^27^. The above four shRNAs (shRNA-Gal-3-372, shRNA-Gal-3-556, shRNA-Gal-3-444, and shRNA-Gal-3-738) and the negative control shRNA (shRNA-NC) were transfected into passage four to five BM-MSCs using X-tremeGENE HP DNA transfection reagent following the manufacturer’s protocol. After 24 h, the optimal RNA interference vector (shRNA-Gal-3-372) was identified by qPCR (Supplementary Fig. S2). The linearized shRNA-Gal-3-372 vector (Supplementary Fig. S3) or shRNA-NC was transfected into BM-MSCs. Stably transfected cell lines were obtained in culture medium containing G418 (200 μg/ml). The culture medium was changed every 2 days Selection was performed for 14 days.

A lentiviral overexpression vector (Supplementary Fig. S4) was designed and synthesized based on the miniature pig Gal-3 gene sequence by Shanghai GenePharma Co., Ltd. A negative control was also produced according to the manufacturer’s protocol. The virus titer was 1 × 10^9^. Lentivirus-Gal-3 or lentivirus-NC was transduced into BM-MSCs. Stably transfected cell lines were obtained in culture medium containing puromycin (4 μg/ml). The culture medium was changed every 2 days. Selection was performed for about 1 week.

Gal-3 expression in transfected cell lines was examined by qPCR and western blotting. To detect transfection efficiency, the vectors carried the green fluorescent protein gene.

### qPCR analysis

RNA was extracted using an RNA Extraction Kit (Bioteke). First-strand cDNA was synthesized by a Reverse Transcription Kit (Thermo Scientific). qPCR analysis of Gal-3 was performed using SYBR green PCR master mix (Applied Biosystems). The primers were as follows: GAPDH-forward: 5′-AGGGCATCCTGGGCTACACT-3′, GAPDH-reverse: 5′-TCCACCACCCTGTTGCTGTAG-3′; Gal-3-forward: 5′-GGTTGCGGTCAATGATGCTC-3′, Gal-3-reverse: 5′-AGTGTGTGAAGCACTGGTGA-3′.

### Western blot analysis

BM-MSCs were collected and lysed in RIPA buffer (Invitrogen). The protein concentration was measured using a BCA protein Assay Kit (Thermo Scientific). Equal amount of proteins (20 μg) were separated by 10% sodium dodecyl sulfate-polyacrylamide gel electrophoresis (SDS-PAGE) and transferred to a polyvinylidene fluoride membrane. The membranes were blocked in 5% milk powder and then incubated with primary and secondary antibodies. Finally, immunoreactive bands were developed by enhanced chemiluminescence (Thermo Scientific). Primary antibodies were against Gal-3 (1:750, abcam), Erk (1:1000, Cell Signaling Technology), p-Erk1/2 (1:1000, Cell Signaling Technology), Akt (1:1000, Cell Signaling Technology), p-Akt (ser473) (1:1000, Cell Signaling Technology), and β-actin (1:1000, Cell Signaling Technology).

### Cell proliferation assay

BM-MSCs were cultured in 96 well-plates (6 × 10^3^ per well) and their proliferation was analyzed at various time points (1, 2, 3, 4, and 5 days) with a CCK8 kit (Beyotime Biotechnology) following the manufacturer’s protocol.

### Transwell assay

Serum-free medium (200 μl) containing equal numbers of BM-MSCs were added to the upper chambers of a transwell plate (pore size: 8.0 μm; Gibco). The lower chambers contained 800 μl complete medium supplemented with 10% FBS. The cells were incubated for 12 h at 37 °C with 5% CO_2_. Migrated BM-MSCs underneath the membrane were fixed with 4% paraformaldehyde, stained with Hoechst 33342, and then counted under a fluorescence microscope at 20x magnification.

### Wound healing assay

Equal numbers of BM-MSCs were cultured in a 6 well-plate and then treated with 10 μg/ml mitomycin-C for 2 h. A scratch was then made with a 10-μl pipette tip along the middle of the well. Healing of the scratch was photographed under a microscope at various time points (0, 6, 12, 24, 36 and 48 h).

### Detection of Rho family protein activities

BM-MSCs were cultured for 4 h in serum-free medium. The activities of three Rho family proteins, RhoA-, Rac-1- and Cdc42-GTP, were detected using G-LISA kits (Cytoskeleton) following the manufacturer’s protocols.

### Statistical analysis

Results are presented as the mean ± SEM. Statistical analyses were performed by one-way analysis of variance and the Student’s *t*-test. P < 0.05 indicated statistical significance.

## Results

### Establishment of stable Gal-3-knockdown and -overexpressing BM-MSC lines

**Figure 1:**
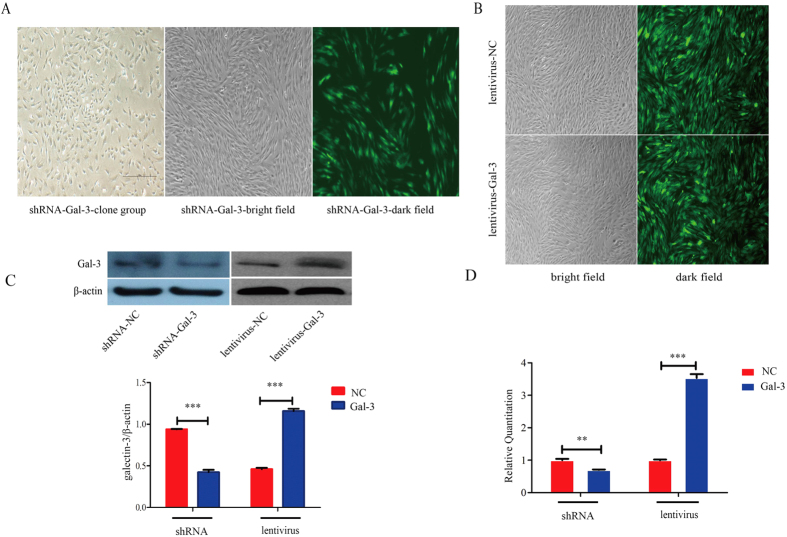
Establishment of stable Gal-3-knockdown and -overexpressing BM-MSC lines. Stable Gal-3-knockdown (**A**) and -overexpressing (**B**) BM-MSCs were confirmed by western blotting and qPCR (**C**,**D**). Magnification: 4x. Error bars represent the standard error of the mean (SEM) (**p < 0.01, ***p < 0.0001).

We established stable cell lines of Gal-3-knockdown and -overexpressing minature pig BM-MSCs. The RNA interference vector was transfected into BM-MSCs. Colonies were observed after selection with G418 for about 7 days, and stable cell lines were obtained after selection for about 14 days (Fig. 1A). Similarly, BM-MSCs were infected with the lentivirus. Stable Gal-3-overexpressing cell lines were obtained after selection with puromycin for about 1 week (Fig. 1B). There were high transfection efficiencies and no apparent effects on morphology. Gal-3 shRNA transfection obviously reduced the expression of Gal-3 at both mRNA (1.00 ± 0.04 in shRNA-NC cells and 0.70 ± 0.02 in shRNA-Gal-3 cells, p = 0.0022) and protein levels. Similarly, Gal-3 lentivirus transduction highly increased the expression of Gal-3 at both mRNA (1.00 ± 0.02 in lentivirus-NC cells and 3.53 ± 0.12 in lentivirus-Gal-3 cells, p < 0.0001) and protein levels (0.9396 ± 0.0030 in shRNA-NC cells and 0.4596 ± 0.1543 in shRNA-Gal-3 cells, p < 0.0001; 0.4242 ± 0.0272 in lentivirus-NC cells and 1.157 ± 0.0293 in lentivirus-Gal-3 cells, p < 0.0001, Fig. 1C,D). After successive passages, the expression status of Gal-3 was stable.

### Gal-3 enhances proliferation and migration of BM-MSCs *in vitro*

**Figure 2:**
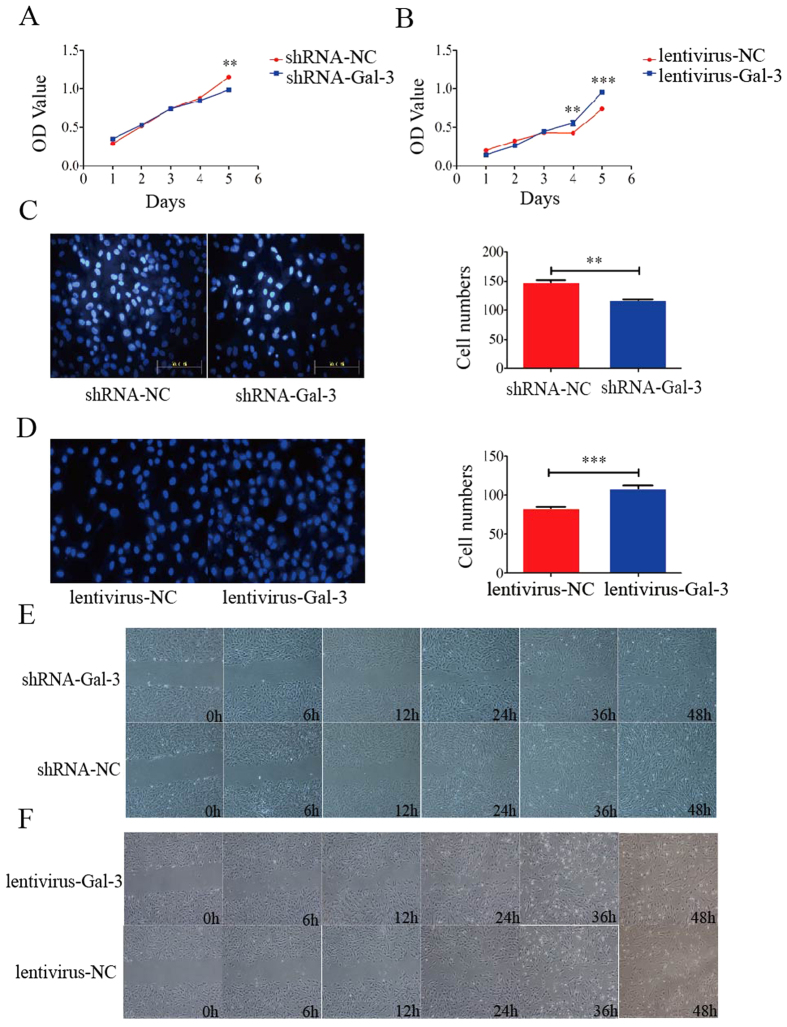
Cell proliferation and migration analyses of Gal-3-knockdown and -overexpressing BM-MSC lines *in vitro*. Gal-3 enhanced the proliferation and migration of BM-MSCs. The proliferation rate of shRNA-Gal-3 cells was lower than that of shRNA-NC cells on day 5 (**A**), and the proliferation rate of lentivirus-Gal-3 cells was higher than that of lentivirus-NC cells on day 4 (**B**). Migrated Gal-3-knockdown (**C**) and -overexpressing (**D**) BM-MSCs in transwell chambers were stained with Hoechst 33342 and quantified (n = 3). Wound healing of Gal-3-knockdown (**E**) and -overexpressing (**F**) BM-MSCs was examined under a microscope. Knockdown of Gal-3 significantly inhibited the migration of BM-MSCs, and overexpression of Gal-3 significantly enhanced the migration of BM-MSCs *in vitro*. Magnifications: 20x (**C**,**D**) and 4x (**E**,**F**). Error bars represent the SEM (**p < 0.01, ***p < 0.0001).

The proliferation assay revealed that Gal-3 enhanced the proliferation of BM-MSCs *in vitro*. We detected the proliferation rate of shRNA-Gal-3 and lentivirus-Gal-3 cell lines, and found that the proliferation rate of shRNA-Gal-3 cells was lower than that of shRNA-NC cells on day 5 (1.15 ± 0.03 in shRNA-NC cells and 0.99 ± 0.03 in shRNA-Gal-3 cells, p = 0.0004) (Fig. 2A),and the proliferation rate of lentivirus-Gal-3 cells was significantly higher than that of lentivirus-NC cells on day 4 (0.43 ± 0.02 in lentivirus-NC cells and 0.56 ± 0.03 in lentivirus-Gal-3 cells, p = 0.004) (Fig. 2B).

Transwell and wound healing assays showed that Gal-3 enhanced the migration of BM-MSCs *in vitro*. In transwell assays, the number of migrated shRNA-Gal-3 BM-MSCs was significantly lower than that of migrated shRNA-NC cells (147.4 ± 4.87 in shRNA-NC cells and 118.0 ± 1.12 in shRNA-Gal-3 cells (n = 3), p = 0.0042) (Fig. 2C), and the number of migrated lentivirus-Gal-3 BM-MSCs was significantly higher than that of migrated lentivirus-NC cells (83.0 ± 2.55 in lentivirus-NC cells and 108.3 ± 4.65 in lentivirus-Gal-3 cells (n = 3), p < 0.0001) (Fig. 2D). Furthermore, wound healing assays showed that the migration speed between the wound edges for shRNA-Gal-3 BM-MSCs was significantly slower than that for shRNA-NC cells after 24 h (Fig. 2E). However, the migration speed between the wound edges for lentivirus-Gal-3 BM-MSCs was significantly faster than that for lentivirus-NC cells after 12 h (Fig. 2F). These data revealed that Gal-3 enhances the proliferation and migration of BM-MSCs *in vitro*.

### Gal-3 enhances the migration of BM-MSCs through inhibition of RhoA-GTP activity

**Figure 3:**
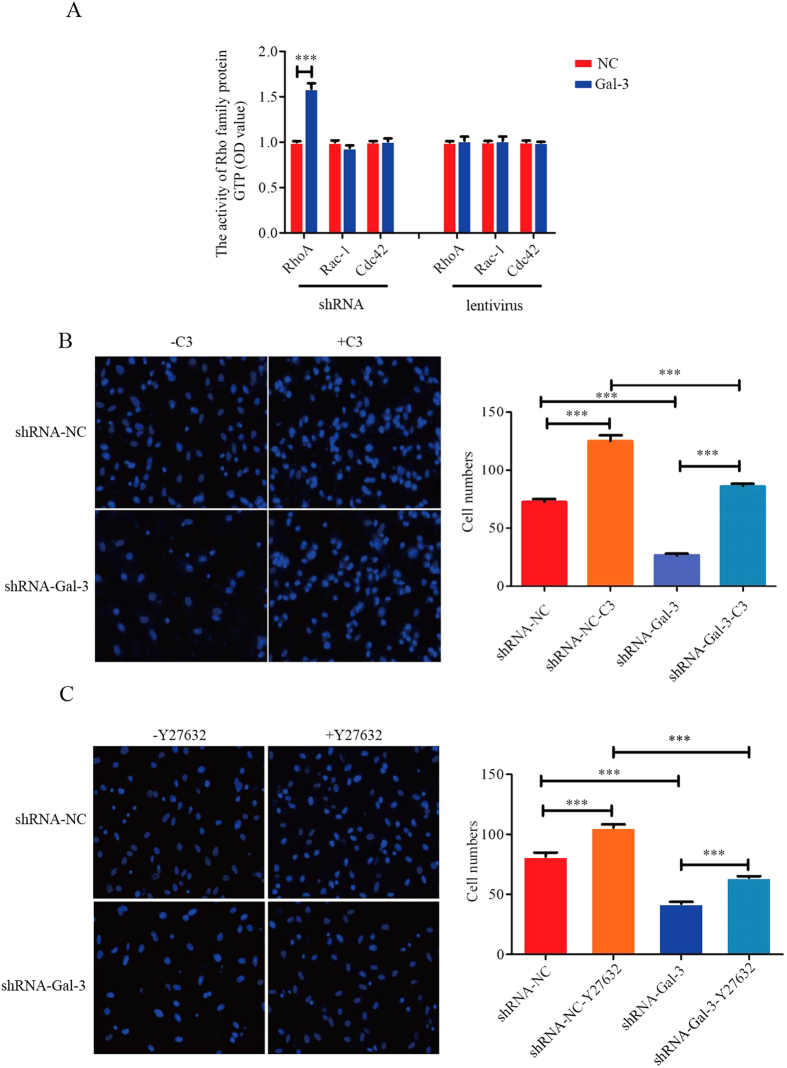
Gal-3 enhances migration of BM-MSCs by inhibition of RhoA-GTP activity. RhoA-, Rac-1- and Cdc42-GTP activities were detected in Gal-3-knockdown and -overexpressing BM-MSCs (**A**). After treatment with C3 transferase (1 μg/ml) for 2 h or Y27632 (10 μM) for 8 h, migrating BM-MSC numbers were quantified in transwell assays (**B**,**C**). The data showed that Gal-3 enhanced cell migration by inhibition of RhoA-GTP activity. Magnification: 20x. Error bars represent the SEM (***p < 0.001).

We detected key regulators of cell migration such as Rho family proteins including RhoA-, Rac-1- and Cdc42-GTPase. RhoA-GTP activity increased in shRNA-Gal-3 BM-MSCs (1.00 ± 0.01 in shRNA-NC cells and 1.59 ± 0.06 in shRNA-Gal-3 cells (n = 3), p < 0.0001), while Rac1- and Cdc42-GTP activities were unchanged. No significant differences in the activities all Rho family proteins were observed in lentivirus-Gal-3 BM-MSCs (Fig. 3A). Therefore, RhoA might be a key regulator of Gal-3 in BM-MSC migration.

To further understand the relationship between RhoA-GTP activity and migration, shRNA-Gal-3 and shRNA-NC BM-MSCs were treated with the RhoA inhibitor C3 transferase or Rho kinase inhibitor Y27632. Transwell assay showed enhancement of shRNA-Gal-3 and shRNA-NC BM-MSC migration compared with untreated cells, and shRNA-Gal-3 BM-MSC migration was lower compared shRNA-NC cells (72.36 ± 2.65 in shRNA-NC cells, 125.0 ± 4.77 in shRNA-NC-C3 cells, 26.67 ± 1.39 in shRNA-Gal-3 cells, and 86.0 ± 2.36 in shRNA-Gal-3-C3 cells; 81.44 ± 3.42 in shRNA-NC cells, 105.6 ± 2.72 in shRNA-NC-Y27632 cells, 42.2 ± 1.61 in shRNA-Gal-3 cells, and 64.0 ± 1.17 in shRNA-Gal-3-Y27632 cells) (Fig. 3B,C). Therefore, Gal-3 enhanced the migration of BM-MSCs through inhibition of RhoA-GTP activity.

### p-Akt (ser473) and p-Erk1/2 are involved in Gal-3-regulated migration of BM-MSCs

To further understand the underlying mechanism of Gal-3 in BM-MSC migration, we detected the expression of p-Akt (ser473) and p-Erk1/2 in both shRNA-Gal-3 and lentivirus-Gal-3 BM-MSCs by western blotting.

**Figure 4:**
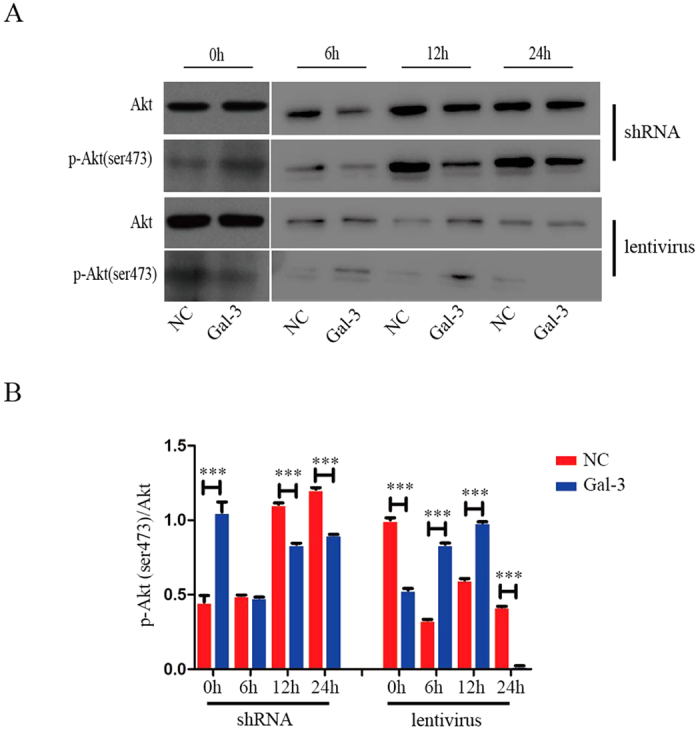
Gal-3 promotes cell migration likely through increasing the expression of p-AKT (ser473). Western blotting was used to analyze the expression of p-Akt (ser473) in Gal-3-knockdown and -overexpressing BM-MSCs (**A**,**B**). The level of p-Akt (ser473) was quantified by ImageJ software. Error bars represent the SEM (***p < 0.001).

**Figure 5:**
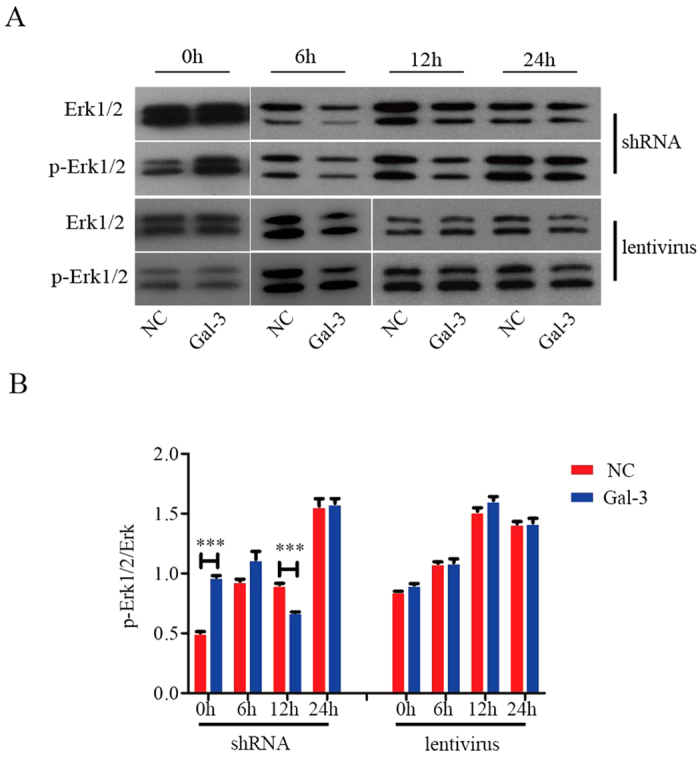
p-Erk1/2 is involved in Gal-3-regulated migration of BM-MSCs. Western blotting was used to analyze the expression of p-Erk1/2 in Gal-3-knockdown and -overexpressing BM-MSCs (**A**,**B**). The level of p-Erk1/2 was quantified by ImageJ software. Error bars represent the SEM (***p < 0.001).

Knockdown of Gal-3 significantly decreased the expression level of p-Akt (ser473) after serum-free starvation for 12 and 24 h. Gal-3 overexpression in BM-MSCs led to an increase in the expression of p-Akt (ser473) in serum-starved cells for 6 and 12 h (Fig. 4). These data indicate that BM-MSC migration stimulated by Gal-3 is likely mediated through increased phosphorylation of AKT (ser473). Next, we found that knockdown of Gal-3 decreased the expression level of p-Erk1/2 in serum-starved BM-MSCs for 12 h. However, no changes were observed in the p-Erk1/2 expression of lentivirus-Gal-3 BM-MSCs, irrespective of serum starvation (Fig. 5). In conclusion, Akt and Erk are involved in Gal-3-regulated migration of BM-MSCs.

## Discussion

**Figure 6:**
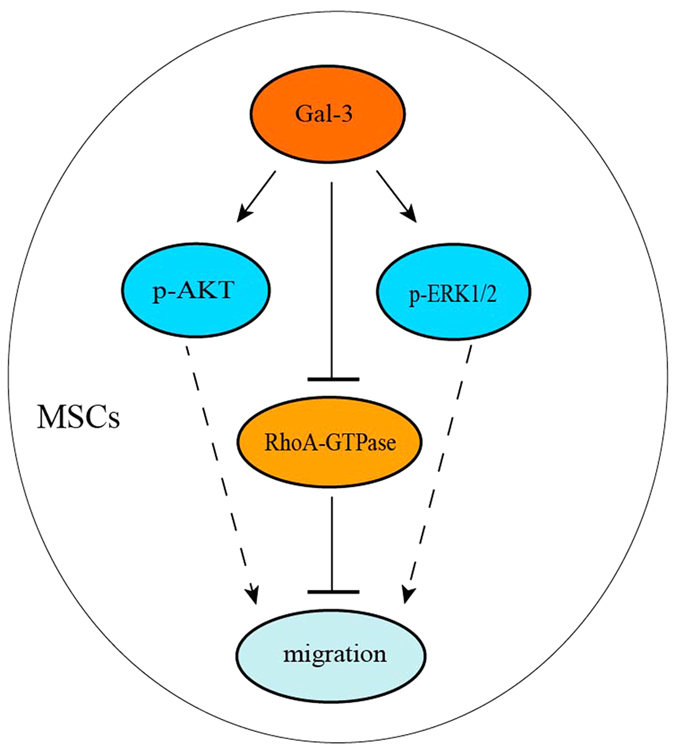
Schematic diagram of the effect of Gal-3 on miniature pig BM-MSC migration. Gal-3 may enhance BM-MSC migration through mainly inhibition of RhoA-GTP activity, increasing p-AKT (ser473) expression, and regulating p-Erk1/2 levels.

Gal-3 has multiple properties and biological activities related to many kinds of diseases^28^,^29^. It is also a regulator of cell migration, and many diseases are related to cell migration^30^, but the exact functions and mechanisms of Gal-3 remain elusive. Enhancement of stem cell migration might contribute to stem cell therapy. Therefore, it would be beneficial for therapeutic applications to understand the function of Gal-3 in regulating BM-MSC migration. Therefore, we established stable Gal-3-knockdown or -overexpressing minature pig BM-MSC lines. Our findings suggest a possible mechanism in which Gal-3 enhances BM-MSC migration through mainly inhibition of RhoA-GTP activity, increasing p-AKT (ser473) expression, and regulating p-Erk1/2 levels (Fig. 6).

Cell proliferation is related to activation of ERK and PI3K/Akt or inhibition of the P38MAPK signaling pathway^21^, which partly supports our data, implying that these signal pathways may be important for minature pig BM-MSC proliferation.

Migration is closely related to various pathological processes involved in vascular and chronic inflammatory diseases including multiple sclerosis and cancer. Polar structure establishment, dynamic processes of actin and microtubule polymerization, regulation of spatial and temporal signal transduction, and other aspects of cell migration have been studied in the past few years^31^. Thus, elucidating cell migration mechanisms is important. In our study, we found that galectin-3 enhances wuzhishan miniature pig BM-MSC proliferation and migration through mainly inhibition of RhoA-GTP activity, increasing p-AKT (ser473) expression, and regulating p-Erk1/2 levels.

Rho family proteins, also called Rho GTPases, are guanosine triphosphate (GTP)-binding proteins belonging to the Ras superfamily. The Rho GTPase family plays an important role in regulation of cytoskeleton reorganization^32^ as well as many cellular behaviors including cell growth, differentiation, and migration^33^,^34^. RhoA, Rac-1, and Cdc42 are key regulators in multiple signaling cascades. They mainly regulate morphological changes and the processes of invasion and metastasis in tumor cells^35^. Rac and Cdc42 induce pseudopodia formation, matrix and extracellular matrix degradation^36^, and contribute to cell invasion and metastasis^37^. RhoA is a key member of the Rho GTPase family, which has been identified as an important protein in the regulation of cell migration. RhoA is mainly involved in stress fiber formation and focal adhesion complex assembly. Although the importance of RhoA has been demonstrated in cell migration, the exact mechanism of RhoA signaling in minature pig BM-MSC migration has not been clarified completely. Our results demonstrated upregulation of RhoA-GTP activity in shRNA-Gal-3 BM-MSCs. After treatment with C3 transferase or Y27632, transwell assays showed enhancement of shRNA-Gal-3 and shRNA-NC BM-MSC migration, and shRNA-Gal-3 BM-MSC migration was lower compared with shRNA-NC BM-MSC migration. Based on these data, it is clear that Gal-3 promotes BM-MSC migration through downregulation of RhoA-GTP activity. We also examined the relationship of Rac1- and Cdc42-GTP activities and Gal-3 in cell migration. The results revealed no changes in Gal-3-knockdown or -overexpressing BM-MSCs. Gal-3 enhancement of minature pig BM-MSC migration was not mediated through regulation of Rac1- or Cdc42-GTP activities. Rho regulates several biological behaviors and functions by activating the downstream target molecule Rho kinase. The RhoA/Rho kinase signaling pathway is also associated with various diseases^38^,^39^. Our data showed upregulation of RhoA-GTP activity in shRNA-Gal-3 BM-MSCs. Therefore, we hypothesized that RhoA inhibition might induce migration of BM-MSCs^40^. We detected cell migration by transwell assays after treatment with RhoA inhibitor C3 transferase or the Rho inhibitor Y27632 that is also a Rho kinase inhibitor. The data confirmed our hypothesis. There was no change in RhoA-GTP activity of lentivirus-Gal-3 BM-MSCs. Therefore, Gal-3 may affect BM-MSC migration to some extent via the RhoA/Rho kinase signaling pathway.

Furthermore, we detected the levels of p-Akt (ser473) and p-Erk1/2. Our data identified a relationship between p-Akt (ser473), p-Erk1/2, and Gal-3 in BM-MSC migration. After serum-free incubation for 12 h, phosphorylation of Akt (ser473) was upregulated in lentivirus-Gal-3 BM-MSCs, and BM-MSC migration increased. These data suggest that BM-MSC migration stimulated by Gal-3 is likely mediated through increased phosphorylation of AKT (ser473). However, when lentivirus-Gal-3 BM-MSCs were incubated in serum-free medium for 24 h, p-Akt(ser473) was hardly expressed, possibly leading to suppression of cell migration. The specific mechanisms will require further study. We also found a decrease in Erk1/2 phosphorylation, along with suppressed migration of shRNA-Gal-3 BM-MSCs that were serum starved for 12 h. However, no change was observed in the expression of p-Erk1/2 in lentivirus-Gal-3 BM-MSCs. Therefore, we propose that Gal-3 may enhance minature pig BM-MSC migration by increasing p-AKT (ser473) expression and regulating p-Erk1/2 levels.

In summary, we showed that Gal-3 plays an important role in promoting minature pig BM-MSC proliferation and migration. Gal-3 may enhance BM-MSC migration through mainly inhibition of RhoA-GTP activity, increasing p-AKT (ser473) expression, and regulating p-Erk1/2 levels. These results may lead to potential applications of Gal-3 in cell therapy. Our findings suggests a novel function of Gal-3 in regulating minature pig BM-MSC migration, and Gal-3 may be a potential target in BM-MSCs for clinical applications.

## Additional Information

**How to cite this article**: Gao, Q. *et al*. Galectin-3 Enhances Migration of Minature Pig Bone Marrow Mesenchymal Stem Cells Through Inhibition of RhoA-GTP Activity. *Sci. Rep.* **6**, 26577; doi: 10.1038/srep26577 (2016).

## Supplementary Material

Supplementary Information
