# Supplementary material for: Galectin-3 Enhances Migration of Minature Pig Bone Marrow Mesenchymal Stem Cells Through Inhibition of RhoA-GTP Activity
Source: Sci Rep. 2016 May 24;6:26577. doi: 10.1038/srep26577 (PMC4877579; doi:10.1038/srep26577)
Supplement: Supplementary Information [file srep26577-s1.doc]

**Supplementary Information**

**Title:**

Galectin-3 Enhances Migration of Minature Pig Bone Marrow Mesenchymal Stem Cells Through Inhibition of RhoA-GTP Activity

**Author list:**

Qian Gao, Ying Xia, Lan Liu, Lei Huang, Yang Liu, Xue Zhang, Kui Xu, Jingliang Wei, Yanqing Hu, Yulian Mu, Kui Li


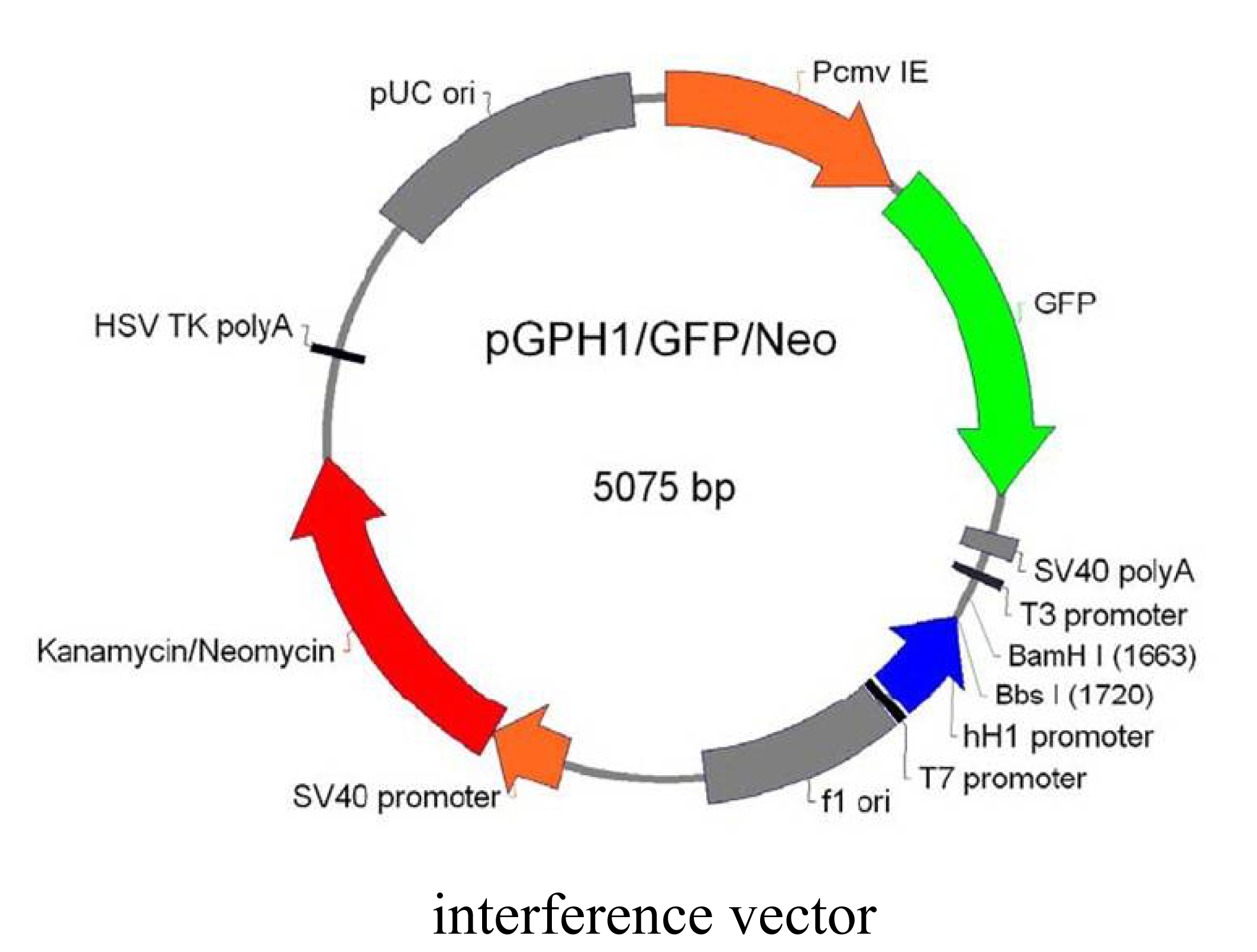


**Figure S1.** **Design of RNA interference vectors.**


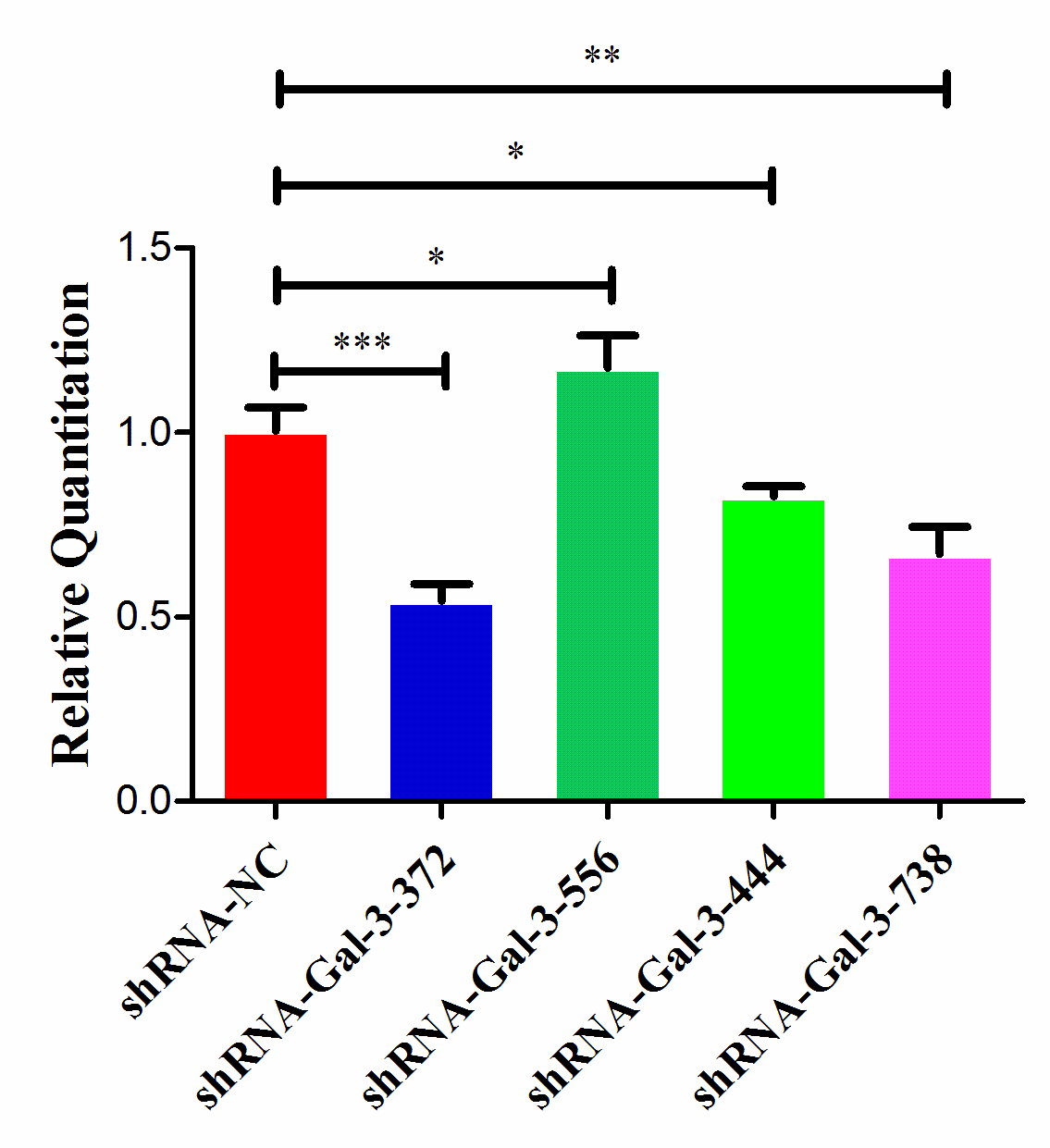


**Figure S2.** **Identification of the optimal RNA interference vector (Galectin-3-372) by qPCR (*p<0.05, **p<0.01, ***p<0.0001).**


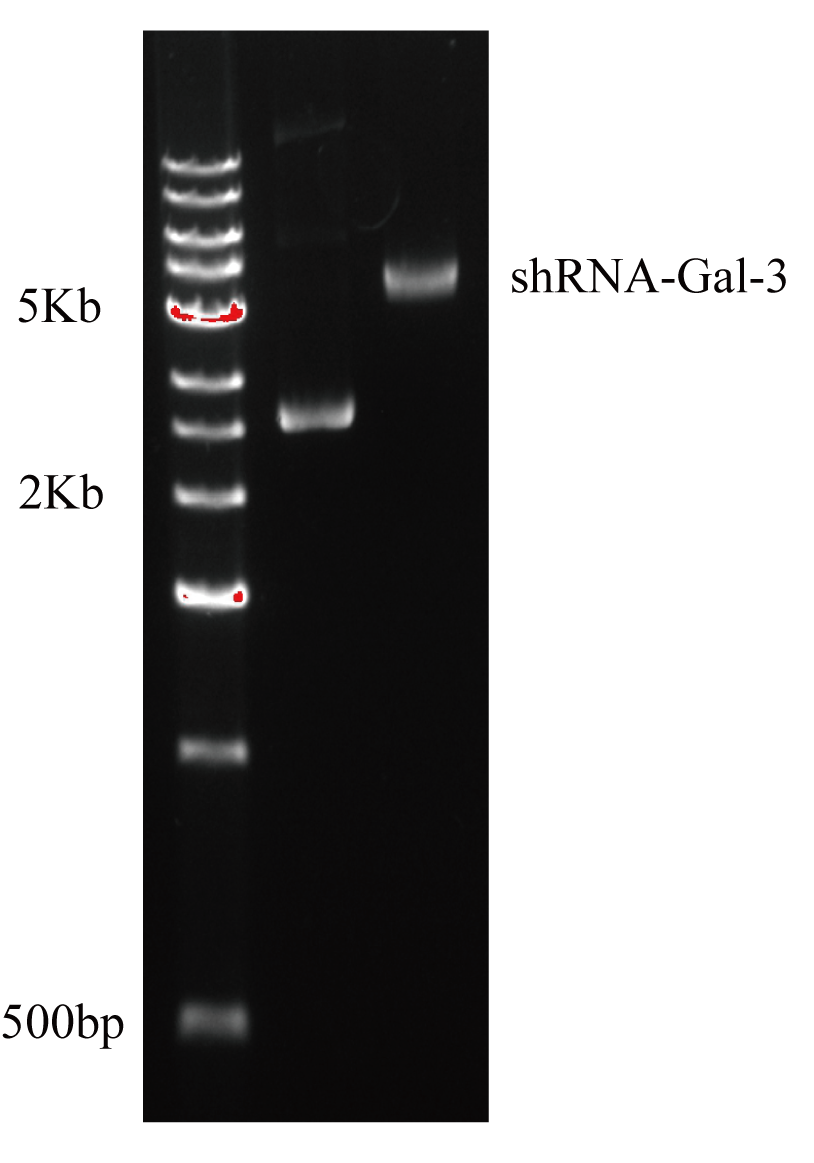


**Figure S3.** **Linearization of the shRNA-Gal-3-372 vector by enzyme digestion.**


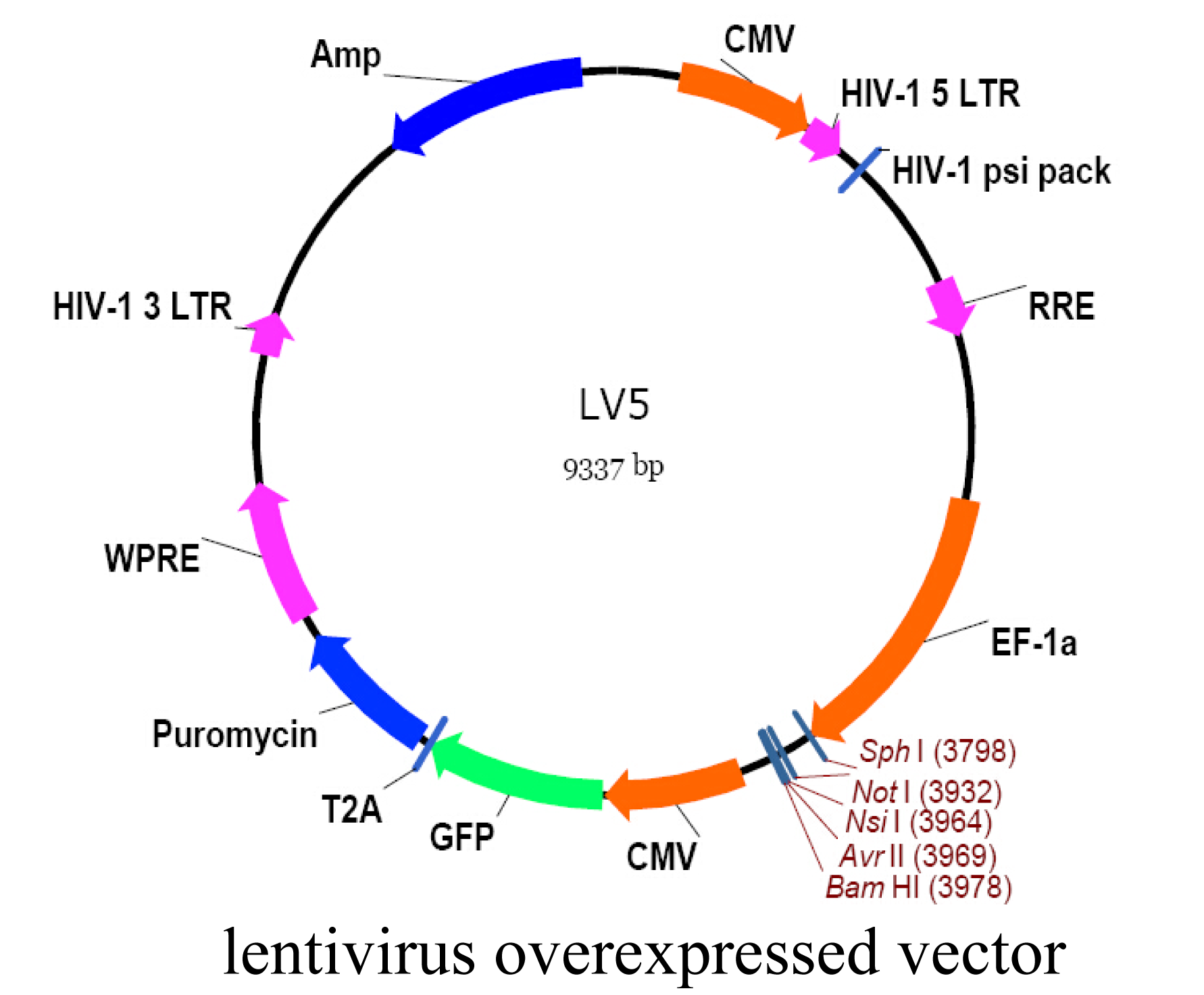


**Figure S4.** **Design of the lentiviral overexpression vector.**
